# Clinical phenotypes and prognostic factors of adult-onset Still’s disease: data from a large inpatient cohort

**DOI:** 10.1186/s13075-021-02688-4

**Published:** 2021-12-08

**Authors:** Rui Li, Xiaolei Liu, Guangliang Chen, Guo Tang, Xiaoxiang Chen, Xuesong Liu, Juan Wang, Liangjing Lu

**Affiliations:** 1 Department of Rheumatology, Renji Hospital, School of Medicine, Shanghai Jiao Tong University, Shanghai, 200127 China; 2 Department of Emergency, Renji Hospital, School of Medicine, Shanghai Jiao Tong University, Shanghai, 200127 China; 3 Department of Medical Oncology, Fudan University Shanghai Cancer Center, Shanghai, 200032 China; 4 Department of Oncology, Shanghai Medical College of Fudan University, Shanghai, 200032 China

**Keywords:** Adult-onset Still’s disease, Clinical phenotypes, Prognosis

## Abstract

**Objectives:**

To define different clinical phenotypes and assess prognostic factors of adult-onset Still’s disease (AOSD).

**Methods:**

Overall, 492 patients with AOSD seen between 2004 and 2018 at a single centre were retrospectively studied.

**Results:**

Of these patients, 78% were female, and the median age at onset was 34 (25–49) years [median (25th–75th percentile)]. The median follow-up time was 7 (3–10) years [median (25th–75th percentile)]. Clinical manifestations at admission were used to subdivide patients with AOSD as follows: systemic inflammation (cluster 1), pure (cluster 2), and intermediate (cluster 3). Each subtype had distinct clinical manifestations and prognoses: cluster 1 (34.6%)—multiple organ manifestations, highest infection rate and mortality, and more than half of the patients with at least one relapse during follow-up; cluster 2 (21.3%)—exclusively female, rash and joint involvement, no internal organ involvement, no mortality, and most of the patients with a monocyclic course; and cluster 3 (44.1%)—less infection rate, no serious complications, and lower mortality rate. The 5- and 10-year survival rates after diagnosis were 92.4% and 86.9%, respectively. Independent risk factors for mortality were age at onset ≥50 (hazard ratio (HR): 6.78, 95% CI: 2.10–21.89), hepatomegaly (HR: 5.05, 95% CI: 1.44–17.70), infection (HR: 15.56, 95% CI: 5.88–41.20), and MAS (HR: 26.82, 95% CI: 7.52–95.60).

**Conclusion:**

Three subtypes of AOSD were identified with distinct clinical manifestations and prognoses. Age at onset ≥50, hepatomegaly, infection, and MAS were prognostic factors for AOSD mortality.

**Supplementary Information:**

The online version contains supplementary material available at 10.1186/s13075-021-02688-4.

## Introduction

Adult-onset Still’s disease (AOSD) is a systemic inflammatory disorder of unknown aetiology. It usually affects young adults and is typically characterized by spiking fever, arthritis, rashes, leucocytosis, and involvement of various organs [1, 2]. The incidence of AOSD has been reported to be 0.16 per 100,000 persons in France [3], 0.22 per 100,000 persons in Japan [4], and 0.4 per 100,000 persons in northern Norway [5]. However, Asian patients are reported to have a significantly higher in-hospital mortality rate [6].

The diagnosis is often delayed because of the low specificity of most findings. At present, the diagnosis of AOSD depends on the exclusion of other diagnoses, such as malignancy, infections, and other rheumatic diseases. The treatment of AOSD is primarily arranged empirically. Nonsteroid anti-inflammatory drugs (NSAIDs), corticosteroids, and synthetic disease-modifying anti-rheumatic drugs (sDMARDs), such as methotrexate (MTX), are generally used as the first-line therapy [7]. In recent years, biological disease-modifying anti-rheumatic drugs (bDMARDs), such as interleukin-1 (IL-1) and interleukin-6 (IL-6) inhibitors, have been successfully used in refractory cases [8]. Despite much recent progress in the management of AOSD, the majority of patients may experience recurrent flares, evolving towards a chronic disease pattern and worse prognosis due to AOSD-related life-threatening complications [9]. Indeed, AOSD is a heterogeneous and complicated disease [1]. Currently, three clinical patterns with acceptable clinical significance [1, 8, 10], i.e., the monocyclic pattern, the polycyclic pattern, and the chronic pattern, are generally identified according to the disease course [11]. However, this classification is not directly based on the clinical features and provides limited information on the management of new-onset AOSD. Recently, the application of data mining techniques using clinical data has been reported to be a promising strategy for understanding the complexity and heterogeneity of some rheumatic diseases and for determining therapeutic approaches and risk stratification [12–16]. The lack of distinguished clinical phenotypes based on the clinical features present at the time of diagnosis as well as the exact report of the long-term survival rate for AOSD based on a large sample size has slowed the progress of precision management of AOSD. Therefore, a better understanding and management of the disease would be possible if the potential clinical models of AOSD were further differentiated.

This study describes our experience with 492 patients with AOSD who were followed up at a single centre from 2004 to 2018. We aimed to define distinct clinical phenotypes and prognostic factors of AOSD.

## Patients and methods

Between January 2004 and December 2018, a total of 492 patients with AOSD who were initially diagnosed and hospitalized in our department at Shanghai Renji Hospital were retrospectively enrolled in this study by review of medical records. We followed up these patients until their death or until September 2019 by telephone or by reviewing outpatient data. A total of 213 (43.3%) patients completed follow-up, and the mean duration of follow-up was 7 (3–10) years [median (25th–75th percentile)] (Supplementary Fig. 1).

All patients fulfilled the diagnostic criteria of AOSD proposed by Yamaguchi M [17]. Table 1 shows the main characteristics of our patients. The assessment at baseline excluded potential mimickers, including infections, cancers, and other autoimmune or autoinflammatory diseases. All clinical/laboratory measurements and therapy were collected at baseline during hospitalization. Pleural effusion and lung parenchymal involvement were evaluated by chest radiograph or computed tomography (CT) imaging. Pericarditis was evaluated by echocardiography. Macrophage activation syndrome (MAS) was defined following the diagnostic criteria proposed by the Histiocyte Society in 1991 and updated in 2004 and by Fardet L. et al. [18–20]. Infection was defined as active bacterial, viral or fungal infection supported by (1) typical clinical and imaging evidence or (2) typical clinical manifestations and positive results of blood, sputum, bone marrow culture or positive results of viral DNA quantitation. A monocyclic pattern was defined as a single episode throughout the entire follow-up period; a relapsing pattern was defined as multiple flares of systemic and/or articular symptoms alternating with disease-free intervals; and a chronic pattern was defined as having persistent symptoms, such as polyarthritis [21]. Refractory AOSD was defined as patients with severe clinical symptoms who had poor responses to high dosages of glucocorticoids or had difficulties withdrawing glucocorticoids even when combined with immunosuppressive agents [22].

**Table 1:** Comparison of clinical features and laboratory test results between the three distinct clusters of AOSD patients

	Total	Systemic inflammation type	Pure type	Intermediate type	*P* value
Number (*n*, %)	492	170 (34.6)	105 (21.3)	217 (44.1)	
Age at onset^a^	37.05±14.34	38.99±14.95	36.03±13.25	36.01±14.27	0.0911
Sex (male/female)	108/384	34/136	0/105	74/143	<0.0001
Typical manifestations (*n*, %)					
Fever ≥ 39 °C	485 (98.6)	167 (98.2)	102 (97.1)	216 (99.5)	0.2108
Rash	417 (84.8)	144 (84.7)	105 (100)	168 (77.4)	<0.0001
Arthralgia	378 (76.8)	124 (72.9)	105 (100)	149 (68.7)	<0.0001
Other clinical findings (*n*, %)					
Pharyngalgia	310 (63)	92 (54.1)	70 (66.7)	148 (68.2)	0.0118
Myalgia	124 (25.2)	43 (25.3)	0 (0)	81 (37.3)	<0.0001
Lymphadenopathy	251 (51)	116 (68.2)	41 (39)	94 (43.3)	<0.0001
Interstitial lung disease	76 (15.4)	76 (44.7)	0 (0)	0 (0)	<0.0001
Pleurisy	60 (12.2)	60 (35.3)	0 (0)	0 (0)	<0.0001
Pericarditis	29 (5.9)	29 (17.1)	0 (0)	0 (0)	<0.0001
Hepatomegaly	33 (6.7)	33 (19.4)	0 (0)	0 (0)	<0.0001
Splenomegaly	140 (28.5)	75 (44.1)	0 (0)	65 (30)	<0.0001
Laboratory findings					
Ferritin > 1500 ng/mL (*n*, %)	348 (70.7)	132 (77.6)	64 (61)	152 (70)	0.0121
WBC (10^9^/L)^a^	17.9±8.2	18.1±9.5	17.4±7.59	17.9±7.2	0.7541
PLT (10^9^/L)^a^	276±107.7	251.6±106.7	295.6±117.7	286±100.1	0.0008
Hb (g/L)^a^	112.7±20.8	111.1±25.1	110.2±16.1	115.2±18.8	0.0568
CRP (mg/L)^a^	87.7±61.6	92.0±64.4	77.0±55.9	89.5±59.1	0.1224
ESR (mm/h)^a^	72.4±34.5	70.4±37.1	74.3±36.8	73.1±31.1	0.6116
ALT (U/L)^a^	318±652.9	483.8±967.9	116.7±160.9	287±425.7	<0.0001
Infection (*n*, %)	55 (11.2)	38 (22.4)	1 (1.0)	16 (7.4)	<0.0001
MAS (*n*, %)	33 (6.7)	33 (19.4)	0 (0)	0 (0)	<0.0001

*WBC* White blood cell, *PLT* Platelet, *Hb* Haemoglobin, *CRP* C-reactive protein, *ESR* Erythrocyte sedimentation rate, *ALT* Alanine transaminase, *MAS* Macrophage activation syndrome

^a^Data are presented as the mean ± S.D.

To identify possible subtypes of AOSD, we performed a cluster analysis of all 492 patients based on the clinical features and laboratory information. Variables such as sex, age, rash, arthralgia, interstitial lung disease (ILD), pharyngalgia, myalgia, pleurisy, pericarditis, hepatomegaly, abnormal liver function test results, splenomegaly, lymphadenopathy, a high level of ferritin (>1500 ng/mL), and MAS were included in the cluster analysis (Supplementary Fig. 1). We fit the model with the latent class analysis (LCA) method using the Gaussian finite mixture model (GMM) clustering algorithm in R software (version 3.6.1) (using the “mclust” package, version 5.4.6). GMM is a kind of probabilistic clustering technique that clusters data points based on the likelihood that they belong to a particular distribution. This means that data points in the same clusters were more similar to other data points in the same cluster and dissimilar to the data points in other clusters. The relative optimal model was chosen based on the Bayesian information criterion (BIC) according to the instructions [23], which would help increase likelihood and avoid overfitting. After that, we used hypothesis testing to compare variables, looking for significant differences between the clusters.

To test the correlation between our classification method and the prognosis of patients with AOSD, the survival of patients who completed the follow-up in each cluster was analysed. Furthermore, 213 patients who completed follow-up were analysed with Cox regression analysis to identify possible prognostic factors of AOSD. All patient baseline clinical characteristics were included in the univariate Cox regression analysis, and variables with *p* <0.05 were then used in the multivariate Cox regression analysis to identify independent risk factors (raw data in Supplementary Table 4).

### Statistical analysis

Continuous variables were compared with *t* tests (for normally distributed data). Categorical variables were compared using the chi-square test or Fisher’s exact test. The GMM clustering algorithm was used and implemented in R software (version 3.6.1) with the “mclust” package (version 5.4.6). Survival data were analysed via Kaplan-Meier plots and compared with the log-rank (Mantel-Cox) test. Statistical results were generated with Prism 8 (8.0.2) software. *P* <0.05 (two tail) was considered significant.

## Results

### Baseline clinical characteristics

Females (78%, *n*=384) predominated in the 492 patients with AOSD. The median age was 34 (25–49) years [median (25th–75th percentile)]. As shown in Table 1, most patients had a high fever (≥39°C) (98.6%, *n*=485), typical skin rash (84.8%, *n*=417), arthralgia (76.8%, *n*=378), pharyngalgia (63%, *n*=310), lymphadenopathy (51.0%, *n*=251), and elevated hepatic enzymes (54.3%, *n*=267) at admission. A few patients also presented with myalgia (25.2%, *n*=124), pleurisy (12.2%, *n*=60), pericarditis (5.9%, *n*=29), ILD (15.4%, *n*=76), hepatomegaly (6.7%, *n*=33) and splenomegaly (28.5%, *n*=140). For inflammatory markers in laboratory testing, a majority of patients (70.7%, *n*=348) showed high serum ferritin levels (>1500 ng/mL). Some life-threatening complications associated with AOSD were also observed, such as macrophage activation syndrome (MAS) (6.7%, *n*=33), disseminated intravascular coagulation (DIC) (0.8%, *n*=4), pulmonary arterial hypertension (PAH) (0.6%, *n*=3), liver failure (0.4%, *n*=2), and fulminant hepatitis (0.2%, *n*=1).

Fifty-five patients presented with infection during hospitalization. Of these, pulmonary infections (7.1%, *n*=35) were the most common type, followed by bone marrow infections (0.6%, *n*=3), sepsis (0.8%, *n*=4), reactivated HBV (0.6%, *n*=3), tuberculosis (0.4%, *n*=2), gastrointestinal fungal infections (0.6%, *n*=3, one coexisting with pulmonary infection), upper respiratory infections (0.8%, *n*=4), and herpes zoster (0.4%, *n*=2).

### Treatment regimen

Almost all patients (99.6%, *n*=490) received glucocorticoids as the initial treatment (Table 2). Table 2 lists the minimum effective equivalent dosage of prednisone needed to achieve a response. As indicated, approximately half of the patients required a high dose of prednisone (>1 mg/kg/day) (47.6%, *n*=234) or a moderate dose of prednisone (0.5–1 mg/kg/day) (46.7%, *n*=229) to achieve an initial remission. However, only a few patients (5.5%, *n*=27) required low-dose corticosteroids (<0.5 mg/kg/day) to achieve remission. Of note, one-fifth of patients (19.3%, *n*=95) achieved clinical remission with glucocorticoid therapy only, without second-line therapy.

**Table 2:** The maximum dosage of glucocorticoids required to achieve rapid remission in AOSD patients

Glucocorticoids (mg/kg/day)	Total (*n*, %)	Systemic inflammation type (*n*, %)	Pure type (*n*, %)	Intermediate type (*n*, %)	*P* value
<0.5	27 (5.5)	6 (3.6)	12 (11.5)	9 (4.1)	0.0097
0.5–1.0	229 (46.7)	59 (34.9)	58 (55.8)	112 (51.6)	0.0006
>1.0	234 (47.6)	104 (61.5)	34 (32.7)	96 (44.2)	<0.0001

*AOSD* Adult-onset Still’s disease

Four-fifth of the patients (80.7%, *n*=397) received immunosuppressants or immunomodulatory agents (Supplementary Table 1). As shown, the most common immunosuppressant was methotrexate (53.5%, *n*=263), followed by hydroxychloroquine (34.3%, *n*=169), cyclosporine (6.3%, *n*=31), thalidomide (4.9%, *n*=24), leflunomide (3.7%, *n*=18), cyclophosphamide (1.2%, *n*=6), tacrolimus (1.0%, *n*=5), mycophenolate mofetil (MMF) (0.2%, *n*=1), and total glucosides of paeony (TGP) (4.1%, *n*=20). In addition, biological agents were additionally administered to patients with refractory or recurrent AOSD (1.6%, n=8). Among these drugs, anti-tumour necrosis factor (TNF) drugs (0.8%, *n*=4) and tocilizumab (0.8%, *n*=4) were the most commonly used. Eleven patients (2.2%) received etoposide (VP-16) for the treatment of MAS. Sixteen patients (3.3%) received intravenous immunoglobulin (IVIG) as adjuvant treatment.

### Clustering analysis

**Fig. 1:**
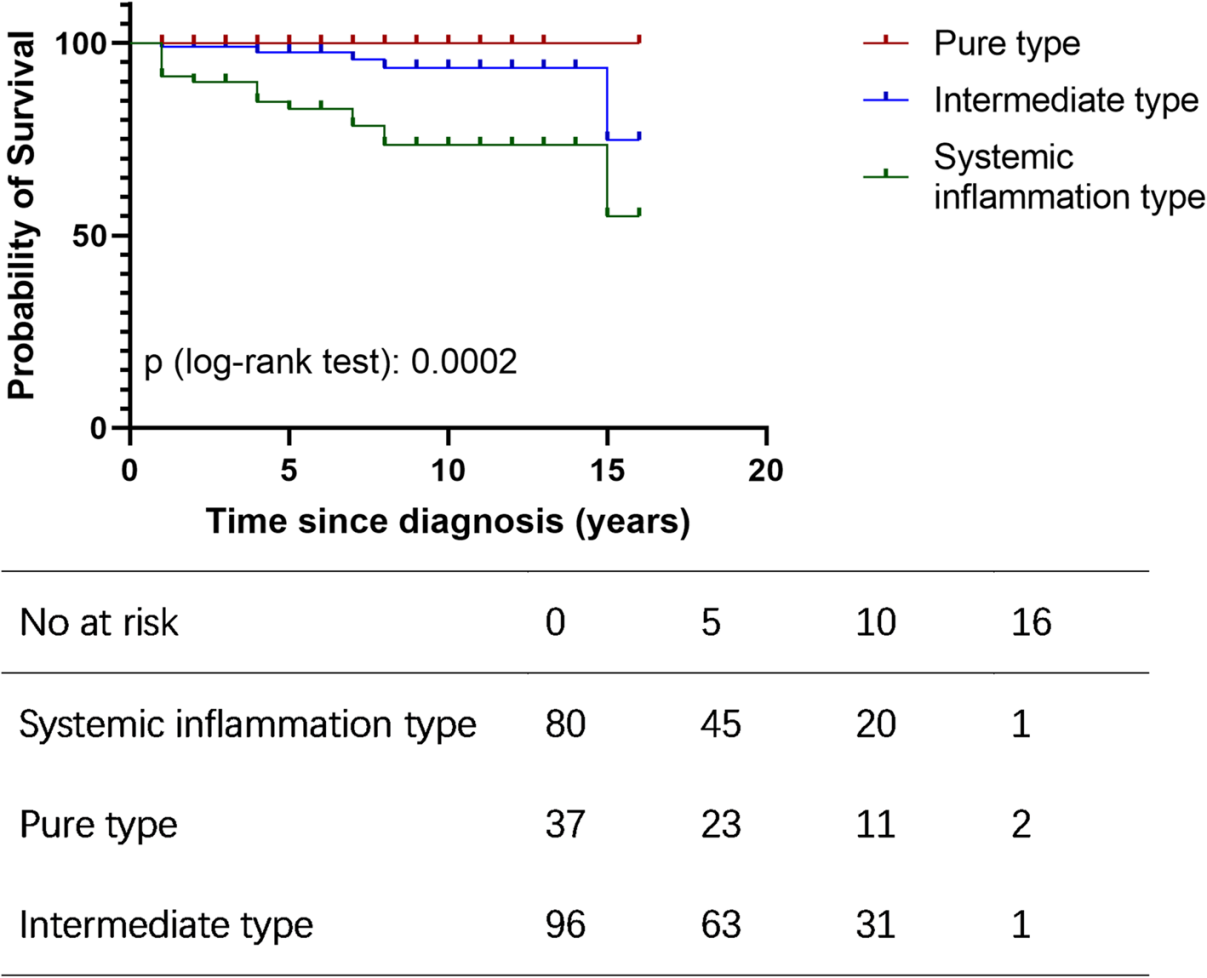
Kaplan-Meier survival curves of three distinct AOSD (adult-onset Still’s disease) subgroups of patients

To identify possible subtypes of the disease, a method of unsupervised clustering of the clinical characteristics was adopted. After fitting candidate cluster models with GMMs, we found that the three-subgroup model had the best fit statistics (Supplementary Fig. 2). Indeed, three subgroups of patients with AOSD were also found to have clinical significance (Table 1). The three groups were defined as the systemic inflammation type (cluster 1), the pure type (cluster 2), and the intermediate type (cluster 3). Moreover, the three subgroups of patients with AOSD were found to be associated with prognosis. The survival of the three subgroups was analysed with Kaplan-Meier survival curves (Fig. 1, Supplementary Table 3).

**Fig. 2:**
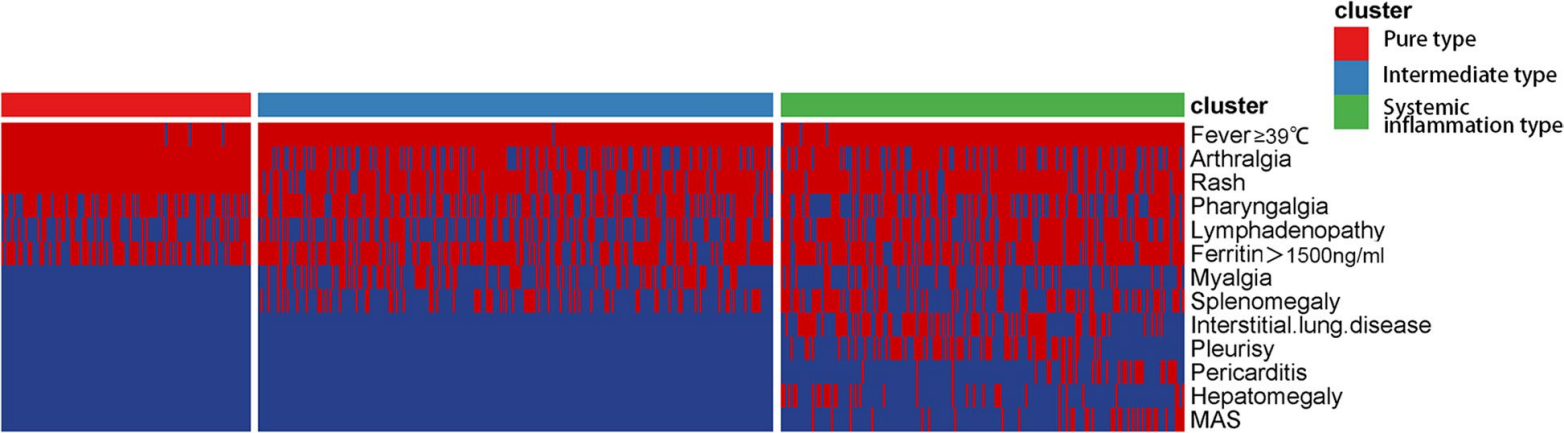
Visualization of clinical manifestations of the three AOSD (adult-onset Still’s disease) subgroups of patients. Each column represents a patient with (red line) or without (blue line) the corresponding symptom of the row

As shown in Table 1 and Fig. 2, patients in cluster 1, with the systemic inflammation type, tended to have multiple organ manifestations, such as lymphadenopathy (68.2%, *n*=116), interstitial lung disease (44.7%, *n*=76), pleurisy (35.3%, *n*=60), pericarditis (17.1%, *n*=29), hepatomegaly (19.4%, *n*=33), and splenomegaly (44.1%, *n*=75). They also had the highest concurrent MAS rate (19.4%, *n*=33) and infection rate (22.4%, *n*=38). Indeed, most patients in cluster 1 received a high dose of steroids (61.5%, >1.0 mg/kg) during hospitalization for remission (Table 2), more than half (58.8%) of the patients experienced at least one relapse during follow-up, and this subgroup had the highest mortality rate (21.3%, *n*=17) (Supplementary Table 2). The 5- and 10-year survival rates after diagnosis were 82.9% and 73.5% (Supplementary Table 3), respectively.

In contrast, patients in cluster 2 had no mortality or MAS occurrence, suggesting a mild disease course. These patients were exclusively female, and all the patients showed rash and joint involvement but no internal organ involvement. A lower dosage of steroids compared with the other clusters was sufficient (55.8%, 0.5–1.0 mg/kg; 11.5%, <0.5 mg/kg) to induce remission (Table 2), and most of these patients presented with a monocyclic course (64.9%) (Supplementary Table 2).

Cluster 3 was the largest group and had an intermediate prognosis. Some of the patients in cluster 3 had splenomegaly and myalgia in addition to general symptoms, but they did not develop MAS. The mortality rate of cluster 3 (5.2%) was significantly lower than that of cluster 1 (21.3%) (Supplementary Table 2). The 5- and 10-year survival rates after diagnosis were 97.5% and 93.6% (Supplementary Table 3), respectively.

### Prognosis

In our cohort, a total of 213 (43.4%) patients completed follow-up. The median follow-up time was 7 (3–10) years [median (25th–75th percentile)], and the longest follow-up time was 16 years. During the follow-up period, half of the patients had monocyclic disease (54.0%, 115/213), while the rest had relapsing or chronic erosive patterns (46.0%, 98/213) (Supplementary Table 2). Twenty-two patients (10.33%, 22/213) ultimately died. The 5-year survival rate and 10-year survival rate after diagnosis were 92.4% and 86.9%, respectively.

Both univariate and multivariate Cox regression were used to predict AOSD patient mortality from a number of clinical and laboratory measurements at baseline (Table 3). Multivariate analysis was performed using the following variables with *p* <0.05 in univariate analysis: age at onset ≥50, ILD, infection, pericarditis, pleurisy, MAS and hepatomegaly. The results of multivariate analysis showed that the independent risk factors for mortality were age at onset ≥50 (hazard ratio (HR): 6.78, 95% CI: 2.10–21.89), hepatomegaly (HR: 5.05, 95% CI: 1.44–17.70), infection (HR: 15.56, 95% CI: 5.88–41.20) and MAS (HR: 26.82, 95% CI: 7.52–95.60).

**Table 3:** Cox regression was used to analyse the correlation between baseline measurements and the survival of AOSD patients

	Univariate analysis		Multivariate analysis	
Variables	Hazard ratio (95% CI)	*p*	Hazard ratio (95% CI)	*p*
Age at onset, years (continuous)	1.03 (1.00–1.06)	0.026		
Age at onset, years (categorical)				
<50	0.31 (0.13–0.73)	0.008		
≥50	3.20 (1.36–7.53)	0.008	6.78 (2.10–21.89)	0.001
Gender				
Male	1.32 (0.51–3.36)	0.567		
Female	0.76 (0.30–1.95)	0.567		
Fever ≥ 39 °C	-	0.997		
Rash	0.59 (0.22–1.59)	0.292		
Arthralgia	0.66 (0.27–1.61)	0.358		
ILD	2.92 (1.22–6.98)	0.016	0.52 (0.13–2.14)	0.369
Pharyngalgia	1.29 (0.52–3.17)	0.581		
Myalgia	1.41 (0.55–3.59)	0.478		
Pericarditis	3.76 (1.23–11.4)	0.020	1.17 (0.36–3.78)	0.792
Pleurisy	3.04 (1.27–7.26)	0.012	2.17 (0.59–8.03)	0.246
Hepatomegaly	4.14 (1.50–11.38)	0.006	5.05 (1.44–17.70)	0.011
Splenomegaly	1.49 (0.62–3.55)	0.371		
Lymphadenopathy	1.16 (0.50–2.68)	0.734		
Ferritin > 1500 ng/mL	2.04 (0.69–6.01)	0.198		
Infection	12.56 (5.39–29.28)	4.52e^−09^	15.56 (5.88–41.20)	3.29e^−08^
MAS	11.88 (4.51–31.30)	5.58e^−07^	26.82 (7.52–95.60)	3.95e^−07^

*ILD* Interstitial lung disease, *MAS* Macrophage activation syndrome

## Discussion

We reported a recent and large series of patients with AOSD and described clinical and laboratory features, treatment regimens, and disease evolution parameters over a long period, 2004–2018. We first analysed the clinical and laboratory features of the large cohort of 492 patients with AOSD and found that patients with AOSD could be subdivided into three subtypes, the systemic inflammation type, pure type, and intermediate type, which had distinct clinical manifestations and prognoses. In addition, age at onset ≥50, infection, hepatomegaly and MAS were independent risk factors for predicting AOSD mortality.

Our study demonstrated that our AOSD cohort was similar in mean age and sex distribution to those in prior reports [24]. In our cohort of 492 patients with AOSD, 384 patients were female, and the median age at onset was 34 (25–49) years [median (25th–75th percentile)]. The median follow-up time was 7 (3–10) years [median (25th–75th percentile)]. The typical clinical features included the classic triad of fever, arthritis and rash characteristic of AOSD. Corticosteroid therapy is used as the first-line treatment for AOSD. Consistent with other reports [9, 25], the induction of remission was mostly achieved with moderate/high doses of corticosteroids and/or DMARD combinations, including methotrexate and hydroxychloroquine. If MTX fails to control the disease, other conventional synthetic DMARDs may be taken into consideration. Remission was achieved in the majority of patients using this treatment scheme. It has been proven that TNF-α, IL-1 and IL-6 blockers are effective in AOSD, and IL-6 blockade could be more effective in the control of arthritic manifestations, whereas IL-1 could be a better target in cases of predominant systemic manifestations [21, 26]. Only 1.6% of patients required biological agents to control refractory disease or recurrence in our cohort, which was lower than the other published cohorts [9, 21]. It was related to the economic level, and the drugs were used off label. For recurrent and refractory cases, we have used more biological agents in recent years.

According to the Yamaguchi criteria [17], the three major typical manifestations of AOSD are spiking fever, typical rash and arthralgia. In our cohort, spiking fever was a common feature of the three types. However, only patients with the pure type of AOSD had both arthralgia and skin rash, while the other two types of patients did not necessarily have these two manifestations at the same time. Patients with systemic inflammation had slightly lower rates of rash and joint pain, and they had the highest probabilities of visceral involvement, including ILD, pleurisy, pericarditis, and hepatosplenomegaly, as well as life-threatening MAS. Patients with the pure type did not have serious complications affecting internal organs but did have sore throat, large lymph nodes, and mildly elevated liver transaminase levels. Intermediate-type patients, similar to systemic inflammatory patients, also had lower rates of rash and joint pain but had limited visceral involvement (no cardiopulmonary issues, hepatosplenomegaly or MAS).

There are some differences between our clustering and previous studies. Several studies have shown that AOSD can be divided into systemic subtype and chronic articular subtype. For the systemic subtype, multi-organ involvement and high probabilities of MAS occurrence [11, 27], similar to the systemic inflammation type in our study. The chronic articular subtype exhibits a pre-eminence of chronic polyarthritis, with a lower inflammatory state but possible joint destruction and is thus considered rheumatoid arthritis (RA) [11, 27]. Moreover, it has been reported that female sex, proximal arthritis at disease onset and steroid dependence may predict the chronic articular form of AOSD [21]. In our study, we found that pure-type patients were exclusively female, and all the patients showed rash and joint involvement but no internal organ involvement or mortality or MAS occurrence, which is similar to the chronic articular subtype. However, we found that most of these patients in the pure type presented with monocyclic courses (64.9%), not chronic diseases resembling rheumatoid arthritis. These patients with the pure type having a relatively better prognosis may represent a group of patients with different pathogenesis from the conventional systemic subtype and chronic articular subtype.

In addition to clinical manifestations, the three types of patients had different rates of secondary infection. Patients with systemic inflammation had the highest probability of infection, followed by patients with the intermediate type. Only one infection was found in pure-type patients in our cohort. These different probabilities of infection may be attributed to the different doses of glucocorticoid treatment. Systemic inflammation-type patients received the highest glucocorticoid dosage among patients from all three clusters.

To date, no factors have been identified that can guide physicians regarding an accurate dose of glucocorticoids when a patient is first diagnosed. It was reported that in patients who present an acute flare at admission, a highly active mononuclear phagocyte system and important organ involvement might require a higher dose of glucocorticoids after diagnosis [10]. We found that most patients in cluster 1 (systemic inflammation type) who had multiple organ manifestations received a high dose of steroids. In contrast, patients in cluster 2 (pure type), who had no serious complications and were all female, needed a relatively low dose of steroids to induce remission.

To analyse the prognosis of the three types of patients, we compared the survival conditions and disease recurrence rates. The results showed that patients with the systemic inflammation type of disease had the worst prognosis; the survival rate was significantly lower than that of patients with the pure and intermediate types of disease. In addition, pure-type patients had the highest rate of non-recurrence during follow-up, and systemic inflammatory patients had the highest rate of recurrence. Finally, we predicted that the mortality-associated factors were age at onset ≥50, infection, hepatomegaly, and MAS, which might be valuable for clinical use.

There are several limitations to our study. First, because it is a retrospective study, possible recall and selection errors cannot be excluded. Second, over half of the patients were lost to follow-up, which led to an underestimation of mortality. Third, since only hospitalized AOSD patients were included, the present findings might not be generalizable to all AOSD patients. Finally, the expression of inflammatory factors was diverse in different subtypes, and cytokine profile analysis is needed to further support our clustering [21, 27, 28].

## Conclusions

In this study, we reported a recent series of 492 patients with definite AOSD from a single centre. We highlighted three distinct disease phenotypes. The independent risk factors for predicting AOSD mortality were age at onset ≥50, infection, hepatomegaly and MAS. Additional studies are needed to confirm these findings.

## Supplementary Information

**Additional file 1: Supplementary Figure 1**. Flow chart of patient selection, clustering and follow-up. **Supplementary Figure 2**. Visualization of the three-subgroup model. Each dot represents a patient, and each colour represents a cluster. **Supplementary Table 1**. Comparison of second-line treatments used in different clusters of AOSD patients. **Supplementary Table 2**. Comparison of the prognosis of the three distinct AOSD patient clusters. **Supplementary Table 3**. Survival of the three subgroups patients. **Supplementary Table 4**. Raw data of patients completed follow-up.

## Data Availability

The raw data used during the current study are available from the corresponding author on reasonable request.

## Abbreviations

AOSD: Adult-onset Still’s disease
MAS: Macrophage activation syndrome
ILD: Interstitial lung disease
RA: Rheumatoid arthritis
IL-1: Interleukin-1
IL-6: Interleukin-6
NSAIDs: Nonsteroid anti-inflammatory drugs
sDMARDs: Synthetic disease-modifying anti-rheumatic drugs
MTX: Methotrexate
LCA: Latent class analysis
HR: Hazard ratio
GMM: Gaussian finite mixture model
BIC: Bayesian information criterion
HBV: Hepatitis B virus
PAH: Pulmonary arterial hypertension
DIC: Disseminated intravascular coagulation
MMF: Mycophenolate mofetil
TGP: Total glucosides of paeony
TNF: Anti-tumour necrosis factor
VP-16: Etoposide
IVIG: Intravenous immunoglobulin
WBC: White blood cell
PLT: Platelet
Hb: Haemoglobin
CRP: C-reactive protein
ESR: Erythrocyte sedimentation rate
ALT: Alanine transaminase

## References

[1] Efthimiou P, Paik PK, Bielory L (2006). Diagnosis and management of adult onset Still’s disease. Ann Rheum Dis.

[2] Kontzias A, Efthimiou P (2008). Adult-onset Still’s disease: pathogenesis, clinical manifestations and therapeutic advances. Drugs.

[3] Magadur-Joly G, Billaud E, Barrier JH, Pennec YL, Masson C, Renou P, Prost A (1995). Epidemiology of adult Still’s disease: estimate of the incidence by a retrospective study in west France. Ann Rheum Dis.

[4] Wakai K, Ohta A, Tamakoshi A, Ohno Y, Kawamura T, Aoki R, Kojima M, Lin Y, Hashimoto S, Inaba Y (1997). Estimated prevalence and incidence of adult Still’s disease: findings by a nationwide epidemiological survey in Japan. J Epidemiol.

[5] Evensen KJ, Nossent HC (2006). Epidemiology and outcome of adult-onset Still’s disease in Northern Norway. Scand J Rheumatol.

[6] Mehta BY, Ibrahim S, Briggs W, Efthimiou P (2019). Racial/Ethnic variations in morbidity and mortality in adult onset Still’s disease: an analysis of national dataset. Semin Arthritis Rheum.

[7] Ruscitti P, Cipriani P, Liakouli V, Guggino G, Carubbi F, Berardicurti O, Ciccia F, Giacomelli R (2019). Managing adult-onset Still’s disease: the effectiveness of high-dosage of corticosteroids as first-line treatment in inducing the clinical remission. Results from an observational study. Medicine (Baltimore).

[8] Ortiz-Sanjuan F, Blanco R, Calvo-Rio V, Narvaez J, Rubio Romero E, Olive A, Castaneda S, Gallego Flores A, Hernandez MV, Mata C (2014). Efficacy of tocilizumab in conventional treatment-refractory adult-onset Still’s disease: multicenter retrospective open-label study of thirty-four patients. Arthritis Rheum.

[9] Ruscitti P, Cipriani P, Masedu F, Iacono D, Ciccia F, Liakouli V, Guggino G, Carubbi F, Berardicurti O, Di Benedetto P (2016). Adult-onset Still’s disease: evaluation of prognostic tools and validation of the systemic score by analysis of 100 cases from three centers. BMC Med.

[10] Hu QY, Zeng T, Sun CY, Luo CN, Liu S, Ding TT, Ji ZF, Lu A, Yimaiti K, Teng JL (2019). Clinical features and current treatments of adult-onset Still’s disease: a multicentre survey of 517 patients in China. Clin Exp Rheumatol.

[11] Cush JJ, Medsger TA, Christy WC, Herbert DC, Cooperstein LA (1987). Adult-onset Still’s disease. Clinical course and outcome. Arthritis Rheum.

[12] Sun F, Lei Y, Wu W, Guo L, Wang K, Chen Z, Xu W, Wang X, Li T, Zhang X (2019). Two distinct clinical phenotypes of pulmonary arterial hypertension secondary to systemic lupus erythematosus. Ann Rheum Dis.

[13] Dion J, Costedoat-Chalumeau N, Sene D, Cohen-Bittan J, Leroux G, Dion C, Frances C, Piette JC (2016). Relapsing polychondritis can be characterized by three different clinical phenotypes: analysis of a recent series of 142 patients. Arthritis Rheum.

[14] Shimizu J, Yamano Y, Yudoh K, Suzuki N (2017). Organ involvement pattern suggests subgroups within relapsing polychondritis: comment on the article by Dion. Arthritis Rheum.

[15] Iavindrasana J, Cohen G, Depeursinge A, Muller H, Meyer R, Geissbuhler A. Clinical data mining: a review. Yearb Med Inform. 2009;18(01):121–33. 19855885

[16] Wu CT, Lo CL, Tung CH, Cheng HL. Applying data mining techniques for predicting prognosis in patients with rheumatoid arthritis. Healthcare (Basel). 2020;8(2):85. 10.3390/healthcare8020085 PMC7349569 32260259

[17] Yamaguchi M, Ohta A, Tsunematsu T, Kasukawa R, Mizushima Y, Kashiwagi H, Kashiwazaki S, Tanimoto K, Matsumoto Y, Ota T (1992). Preliminary criteria for classification of adult Still’s disease. J Rheumatol.

[18] Filipovich AH. Hemophagocytic lymphohistiocytosis (HLH) and related disorders. Hematol Am Soc Hematol Educ Program. 2009:127–31. 10.1182/asheducation-2009.1.127 20008190

[19] Henter JI, Horne A, Arico M, Egeler RM, Filipovich AH, Imashuku S, Ladisch S, McClain K, Webb D, Winiarski J (2007). HLH-2004: diagnostic and therapeutic guidelines for hemophagocytic lymphohistiocytosis. Pediatr Blood Cancer.

[20] Fardet L, Galicier L, Lambotte O, Marzac C, Aumont C, Chahwan D, Coppo P, Hejblum G (2014). Development and validation of the HScore, a score for the diagnosis of reactive hemophagocytic syndrome. Arthritis Rheum.

[21] Maria AT, Le Quellec A, Jorgensen C, Touitou I, Rivière S, Guilpain P (2014). Adult onset Still’s disease (AOSD) in the era of biologic therapies: dichotomous view for cytokine and clinical expressions. Autoimmun Rev.

[22] Li T, Gu L, Wang X, Guo L, Shi H, Yang C, Chen S (2017). A pilot study on tocilizumab for treating refractory adult-onset Still’s disease. Sci Rep.

[23] Scrucca L, Fop M, Murphy TB, Raftery AE (2016). mclust 5: clustering, classification and density estimation using Gaussian finite mixture models. R J.

[24] Kalyoncu U, Solmaz D, Emmungil H, Yazici A, Kasifoglu T, Kimyon G, Balkarli A, Bes C, Ozmen M, Alibaz-Oner F (2016). Response rate of initial conventional treatments, disease course, and related factors of patients with adult-onset Still’s disease: data from a large multicenter cohort. J Autoimmun.

[25] Zeng T, Zou YQ, Wu MF, Yang CD (2009). Clinical features and prognosis of adult-onset Still’s disease: 61 cases from China. J Rheumatol.

[26] Feist E, Mitrovic S, Fautrel B (2018). Mechanisms, biomarkers and targets for adult-onset Still’s disease. Nat Rev Rheumatol.

[27] Franchini S, Dagna L, Salvo F, Aiello P, Baldissera E, Sabbadini MG (2010). Efficacy of traditional and biologic agents in different clinical phenotypes of adult-onset Still’s disease. Arthritis Rheum.

[28] Inoue N, Shimizu M, Tsunoda S, Kawano M, Matsumura M, Yachie A (2016). Cytokine profile in adult-onset Still’s disease: comparison with systemic juvenile idiopathic arthritis. Clin Immunol (Orlando, Fla).

