# Supplementary material for: Clinical phenotypes and prognostic factors of adult-onset Still’s disease: data from a large inpatient cohort
Source: Arthritis Res Ther. 2021 Dec 8;23:300. doi: 10.1186/s13075-021-02688-4 (PMC8653615; doi:10.1186/s13075-021-02688-4)
Supplement: Supplementary file 1 — Additional file 1: Supplementary Figure 1. Flow chart of patient selection, clustering and follow-up. Supplementary Figure 2. Visualization of the three-subgroup model. Each dot represents a patient, and each colour represents a cluster. Supplementary Table 1. Comparison of second-line treatments used in different clusters of AOSD patients. Supplementary Table 2. Comparison of the prognosis of the three distinct AOSD patient clusters. Supplementary Table 3. Survival of the three subgroups patients. Supplementary Table 4. Raw data of patients completed follow-up. [file 13075_2021_2688_MOESM1_ESM.docx]

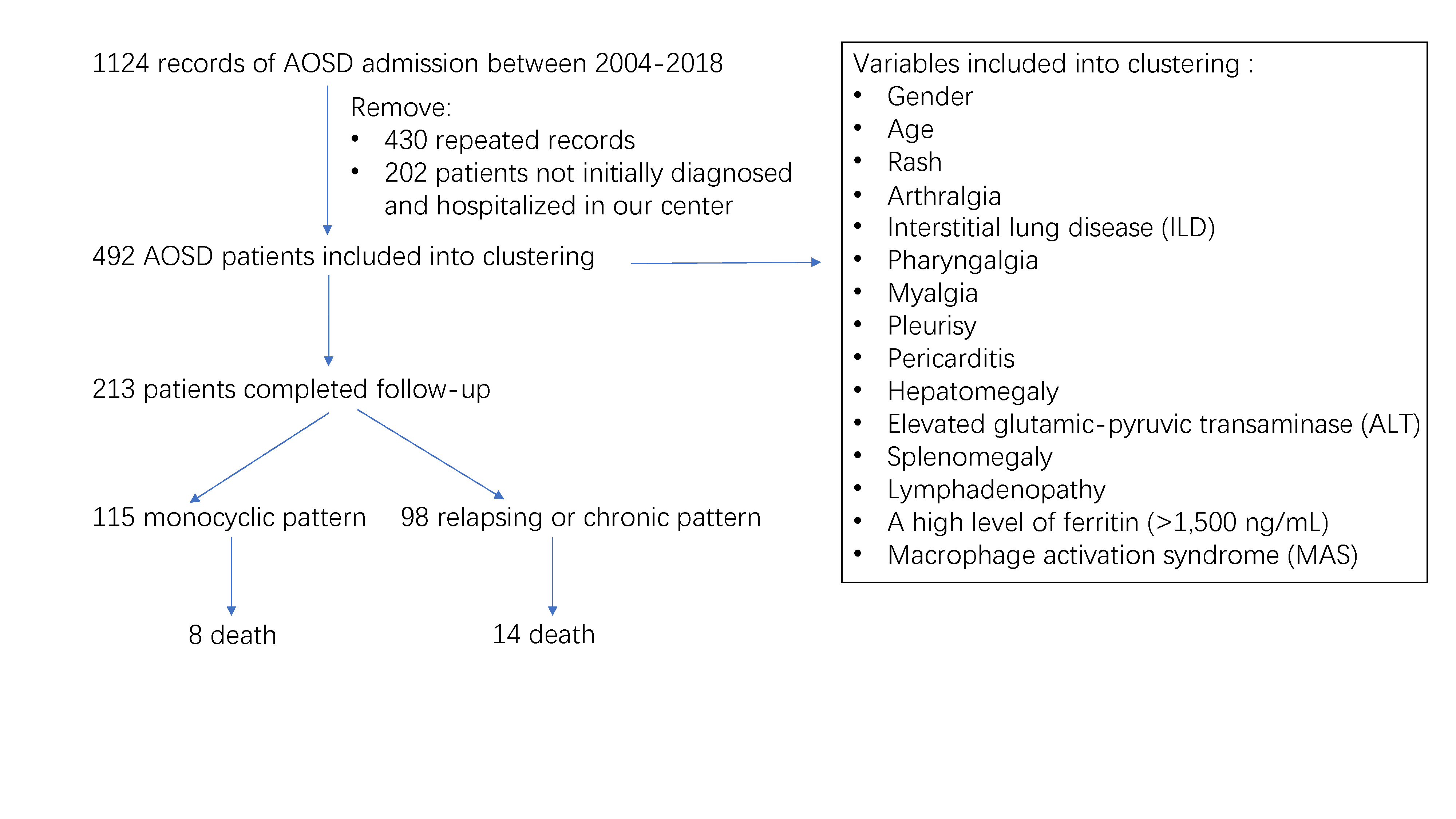


**Supplementary figure 1. Flow chart of patient selection, clustering and follow-up.**


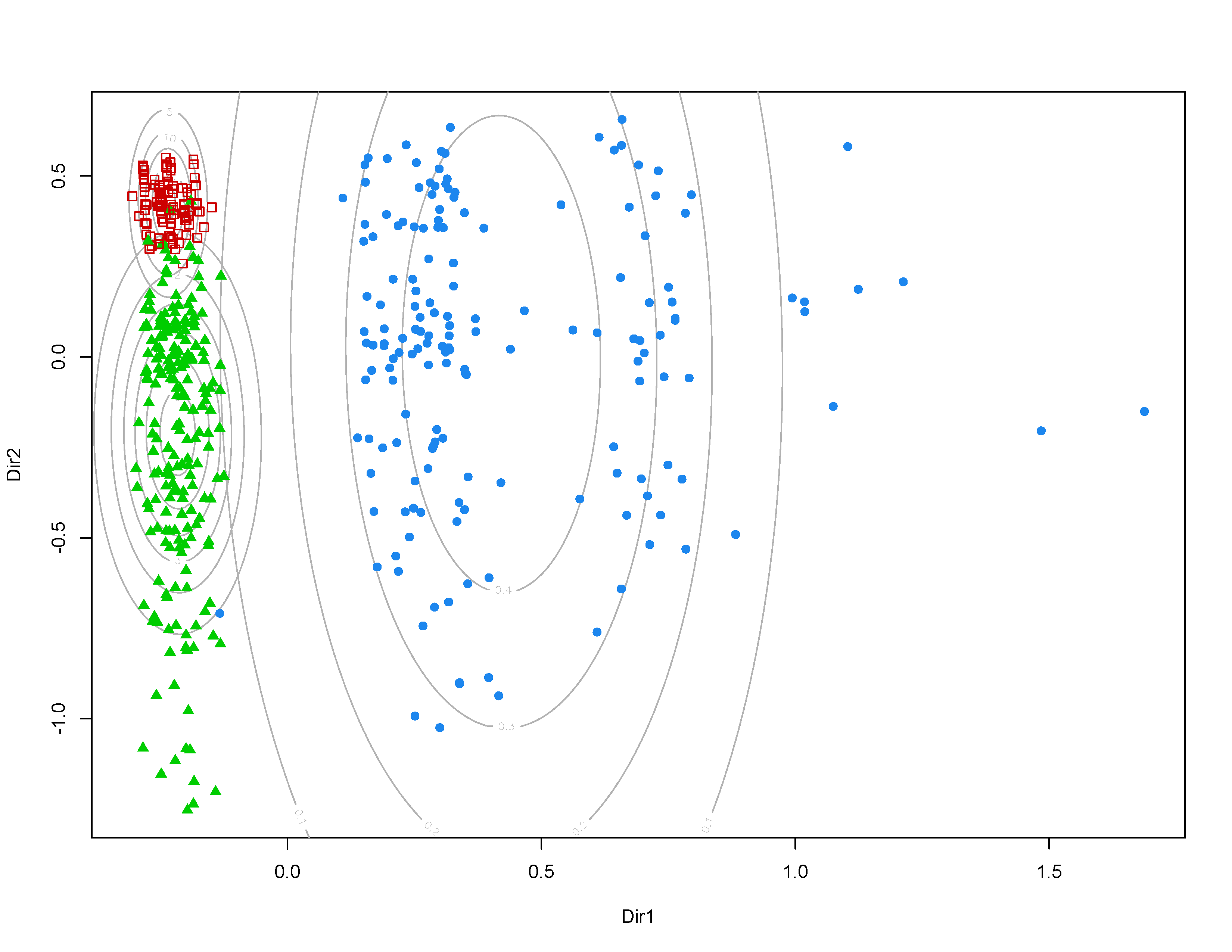
**Supplementary figure 2.** **Visualization of the three-subgroup model. Each dot represents a patient, and each colour represents a cluster.** Blue: cluster 1 (systemic inflammation), red: cluster 2 (pure), green: cluster 3 (intermediate).

**Supplementary table 1. Comparison of second-line treatments used in different clusters of AOSD patients**

| Treatment | Total | Systemic inflammation type | Pure type | Intermediate type | P value |
| --- | --- | --- | --- | --- | --- |
| MTX | 263 (0.53) | 76 (0.45) | 62 (0.59) | 125 (0.58) | 0.0179 |
| HCQ | 169 (0.34) | 47 (0.28) | 48 (0.46) | 74 (0.34) | 0.0091 |
| LEF | 18 (0.04) | 4 (0.02) | 6 (0.06) | 8 (0.04) | 0.3532 |
| AZA | 4 (0.01) | 4 (0.02) | 0 (0) | 0 (0) | 0.0219 |
| CSA | 31 (0.06) | 22 (0.13) | 9 (0.09) | 0 (0) | <0.0001 |
| MMF | 1 (0) | 1 (0.01) | 0 (0) | 0 (0) | 0.3871 |
| FK506 | 5 (0.01) | 2 (0.01) | 0 (0) | 2 (0.01) | 0.5569 |
| CTX | 6 (0.01) | 2 (0.01) | 1 (0.01) | 4 (0.02) | 0.5186 |
| Thalidomide | 24 (0.05) | 6 (0.04) | 10 (0.10) | 8 (0.04) | 0.0447 |
| TGP | 20 (4.1) | 7 (4.1) | 3 (2.9) | 10 (4.6) | 0.7564 |
| NSAIDs | 15 (0.03) | 5 (0.03) | 3 (0.03) | 7 (0.03) | 0.9789 |
| SASP | 5 (0.01) | 1 (0.01) | 2 (0.02) | 2 (0.01) | 0.5619 |
| IVIG | 16 (0.03) | 11 (0.06) | 0 (0) | 5 (0.02) | 0.0076 |
| VP-16 | 11 (0.02) | 11 (0.06) | 0 (0) | 0 (0) | <0.0001 |
| Tocilizumab | 4 (0.01) | 2 (0.01) | 0 (0) | 2 (0.01) | 0.5569 |
| TNF-a inhibitor | 4 (0.01) | 4 (0.02) | 0 (0) | 0 (0) | 0.0219 |

AOSD, adult-onset Still’s disease; MTX, methotrexate, HCQ, hydroxychloroquine; LEF, leflunomide; AZA, azathioprine; CSA, corticosteroids; MMF, mycophenolate mofetil; FK-506, tacrolimus; CTX, cyclophosphamide; NSAIDs, nonsteroidal anti-inflammatory drugs; SASP, sulfasalazine; IVIG, intravenous immunoglobulin; VP-16, etoposide; TNF-a, tumour necrosis factor-a; TGP: total glucosides of paeony.

**Supplementary table 2. Comparison of the prognosis of the three distinct AOSD patient clusters**

|  | **Total** | **Systemic inflammation type** | **Pure type** | **Intermediate type** | **P value** |
| --- | --- | --- | --- | --- | --- |
| Monocyclic disease pattern (n, %) | 115 (54.0) | 33 (41.3) | 24 (64.9) | 58 (60.4) | 0.0137 |
| Relapsing or chronic pattern (n, %) | 98 (46.0) | 47 (58.8) | 13 (35.1) | 38 (39.6) |  |
| Death | 22 (10.3) | 17 (21.3) | 0 (0.0) | 5 (5.2) | 0.0002 |
| Follow-up years* | 6.8±4.4 | 6.3±4.4 | 7.0±4.3 | 7.1±4.4 | 0.9236 |
| Follow-up rate | 43.30% | 47.10% | 35.20% | 44.20% | 0.1469 |

AOSD, adult-onset Still’s disease. *Data are presented as the mean ± S.D.

**Supplementary table 3. Survival of the three subgroups patients.**

| cluster 1 systemic inflammation type | | | | |
| --- | --- | --- | --- | --- |
| Time Since Diagnosis, Years | Number at Risk (Nt) | Number of Deaths  (Dt) | Number Censored  (Ct) | Survival Probability  S_t+1_=S_t_*(N_t+1_-D_t+1_)/N_t+1_ |
| 0 | 80 | 0 | 0 | 1 |
| 1 | 80 | 7 | 8 | 0.913 |
| 2 | 65 | 1 | 7 | 0.898 |
| 3 | 57 | 0 | 4 | 0.898 |
| 4 | 53 | 3 | 5 | 0.848 |
| 5 | 45 | 1 | 3 | 0.829 |
| 6 | 41 | 0 | 3 | 0.829 |
| 7 | 38 | 2 | 5 | 0.785 |
| 8 | 31 | 2 | 5 | 0.735 |
| 9 | 24 | 0 | 4 | 0.735 |
| 10 | 20 | 0 | 5 | 0.735 |
| 11 | 15 | 0 | 4 | 0.735 |
| 12 | 11 | 0 | 2 | 0.735 |
| 13 | 9 | 0 | 1 | 0.735 |
| 14 | 8 | 0 | 4 | 0.735 |
| 15 | 4 | 1 | 2 | 0.551 |
| 16 | 1 | 0 | 1 | 0.551 |

| Cluster 2 pure type | | | | |
| --- | --- | --- | --- | --- |
| Time Since Diagnosis, Years | Number at Risk (Nt) | Number of Deaths  (Dt) | Number Censored  (Ct) | Survival Probability  S_t+1_=S_t_*(N_t+1_-D_t+1_)/N_t+1_ |
| 0 | 37 | 0 | 0 | 1 |
| 1 | 37 | 0 | 2 | 1 |
| 2 | 35 | 0 | 3 | 1 |
| 3 | 32 | 0 | 5 | 1 |
| 4 | 27 | 0 | 4 | 1 |
| 5 | 23 | 0 | 3 | 1 |
| 6 | 20 | 0 | 2 | 1 |
| 7 | 18 | 0 | 4 | 1 |
| 8 | 14 | 0 | 1 | 1 |
| 9 | 13 | 0 | 2 | 1 |
| 10 | 11 | 0 | 2 | 1 |
| 11 | 9 | 0 | 2 | 1 |
| 12 | 7 | 0 | 1 | 1 |
| 13 | 6 | 0 | 4 | 1 |
| 14 | 2 | 0 | 0 | 1 |
| 15 | 2 | 0 | 0 | 1 |
| 16 | 2 | 0 | 2 | 1 |

| Cluster 3 intermediate type | | | | |
| --- | --- | --- | --- | --- |
| Time Since Diagnosis, Years | Number at Risk (Nt) | Number of Deaths  (Dt) | Number Censored  (Ct) | Survival Probability  S_t+1_=S_t_*(N_t+1_-D_t+1_)/N_t+1_ |
| 0 | 96 | 0 | 0 | 1 |
| 1 | 96 | 1 | 10 | 0.99 |
| 2 | 85 | 0 | 9 | 0.99 |
| 3 | 76 | 0 | 8 | 0.99 |
| 4 | 68 | 1 | 4 | 0.975 |
| 5 | 63 | 0 | 7 | 0.975 |
| 6 | 56 | 0 | 3 | 0.975 |
| 7 | 53 | 1 | 6 | 0.957 |
| 8 | 46 | 1 | 9 | 0.936 |
| 9 | 36 | 0 | 5 | 0.936 |
| 10 | 31 | 0 | 8 | 0.936 |
| 11 | 23 | 0 | 5 | 0.936 |
| 12 | 18 | 0 | 6 | 0.936 |
| 13 | 12 | 0 | 3 | 0.936 |
| 14 | 9 | 0 | 4 | 0.936 |
| 15 | 5 | 1 | 3 | 0.749 |
| 16 | 1 | 0 | 1 | 0.749 |
